# Epidemiology and virologic investigation of human enterovirus 71 infection in the Republic of Korea from 2007 to 2012: a nationwide cross-sectional study

**DOI:** 10.1186/s12879-016-1755-0

**Published:** 2016-08-18

**Authors:** Hye-Jin Kim, Ji-Yeon Hyeon, Seoyeon Hwang, Yong-Pyo Lee, Sang Won Lee, Jung Sik Yoo, Byunghak Kang, Jeong-Bae Ahn, Yong-Seok Jeong, June-Woo Lee

**Affiliations:** 1 Division of Vaccine Research, Center for Infectious Diseases, Korea National Institutes of Health, Korea Centers for Disease Control and Prevention, Osong-eup, Cheongju, Chungcheongbuk-do 363-951 Republic of Korea; 2 Department of Biology Graduate School, Kyung Hee University, Seoul, Dongdaemun-gu 130-701 Republic of Korea

**Keywords:** Human enterovirus 71, Hand, Foot and mouth disease, Neurological complications

## Abstract

**Background:**

Enterovirus (EV) 71 is the main pathogen associated with hand, foot and mouth disease (HFMD) or herpangina. Outbreaks of HFMD caused by EV71 infection are associated severe neurological disease and high mortality rates in children. Several sporadic cases of EV71 infection occurred in the Republic of Korea (ROK) in 2000, and EV71 infections were not reported thereafter until 2006. In this prospective study, we report the epidemic and virologic characteristics of the EV71 endemic from 2007 to 2012 in the Republic of Korea.

**Methods:**

We analyzed characteristics of the EV71 infection-associated epidemic from collected specimens and clinical information from 9987 patients with suspected EV infection from the National EV Surveillance System in ROK. To identify the EV71 subgenotype, the homology of viral protein 1 sequences obtained using reverse transcription polymerase chain reaction was compared with the sequences on other countries available from GenBank database.

**Results:**

EV71 was detected in 585 (16.7 %) specimens (cerebrospinal fluid, stool or rectal swabs, throat swabs and blood) during study period and was most frequently observed during epidemic seasons in 2009–2012. Major manifestations due to EV71 infection were HFMD (62.2 %) and HFMD with severe neurological complications (28.4 %). Five deaths (0.9 %) due to EV71 infection occurred, with an increased mortality rate during the period after 2009. Most patients (476; 81.4 %) were less than 5 years of age. Analysis of the monthly distribution showed that there was an obvious seasonal pattern to the epidemics, with infections appearing from June to August. The major subgenotype of EV71 isolates circulating in ROK was the C4a strain, which has also appeared in China, Japan and Vietnam.

**Conclusions:**

This surveillance provided valuable data on the epidemic characteristics of EV71 infections in ROK during a 6-year period. Our findings provide data to assist during future outbreaks of EV71 and associated acute neurologic disease.

## Background

Human enterovirus (EV) 71 belongs to the family *Picornaviridae*, genus *Enterovirus*, which includes polioviruses, echoviruses, coxsackieviruses A and B and EV types 68–70 [1]. EV71 can be classified into three genotypes, A, B and C, based on phylogenetic tree analysis using the nucleotide sequence of virus capsid protein (VP) 1 [2]. EV71, as the main pathogen associated with hand, foot and mouth disease (HFMD) or herpangina associated neurological and cardiac complications, may lead to outbreaks of HFMD, which is associated with high mortality rates in children.

EV71 was first described in 1974 after isolation from patients in California in 1969 [3]. Since then, EV71 has been isolated from various locations worldwide, including the United States of America, Australia and Europe, with circulation of different subgenotypes [4–6]. Strains of B5 subtype have caused outbreaks in Asian countries, including Malaysia, Singapore and Taiwan, since 1997 [7, 8]. Since 1998, EV71 infection rates have appeared to increase, particularly for subgenotype C4 in China [9]. Moreover, in the late 1990s, in the Asia-Pacific region frequent EV71-associated HFMD epidemics have occurred, with high incidences of severe neurologic complications and increased fatality rates [10–15].

From studies of EV71 infections, researchers have found that the central nervous system (CNS) is likely the target of the EV71 virus [16]. Since 2008, outbreaks of EV71 infection associated with neurologic involvement, i.e., HFMD with CNS complications, have been reported in the Republic of Korea (ROK) [17, 18]. Moreover, EV71 has emerged as an important target for the upcoming era of poliomyelitis eradication [8]. In the ROK, sporadic cases of EV71 infection occurred in 2000 [19]; however, death or severe effects of EV71 infection were not reported until 2006. Therefore, in this study, we first examined the epidemic and virologic characteristics of the EV71 endemic from 2007 to 2012 in the ROK. We analyzed the etiology of EV71-associated HFMD or herpangina and complications in ROK from 2007 to 2012 and aimed to identify EV71 strains using phylogenetic analysis.

## Methods

### Surveillance system

The Korea Centers for Disease Control and Prevention (KCDC) National EV Surveillance System consists of 180 clinics managed by pediatric physicians (35 primary clinics, 105 secondary hospitals and 40 tertiary hospitals nationwide) and has monitored EV infections since 1993. Participating physicians collected specimens from patients whose illnesses included HFMD, herpangina, meningitis, encephalitis and sepsis, and documented patient age, date of specimen collection, symptoms and suspected diagnosis. Analysis of the specimens, including typing of relevant EV71 and phylogenetic analysis, was carried out at the National Polio Laboratory of KCDC.

### Patients and clinical samples

Specimens and clinical information were collected from patients suspected of having EV infection from 2007 to 2012. Participating clinics provided cerebrospinal fluid (CSF), stool or rectal swabs, throat swabs and blood samples from the patients. Information regarding age, gender and manifestation was collected for all patient samples, and samples were used to confirm presence of EV71.

### Detection of EV71 in clinical samples

Patient specimens were used for EV71 detection and molecular typing. Viral RNA was extracted using magnetic beads (GM-AUTOPREP Kit; Greenmate Co., Seoul, South Korea) according to the manufacturer’s instructions, and the purified viral nucleic acid was processed by using Freedom EVO (Tecan, Männedorf, Switzerland). A highly conserved 5′ noncoding region was the target of a previously described 196-bp region [20]. To determine the EV genotype, conventional reverse transcription polymerase chain reaction (RT-PCR) was carried out for detection of *VP1* using primers designed in a previously study [21]. In the ROK, cell culture and RT-PCR were used for identification of EV strains from 1993 to 2004; after 2005, detection of *VP1* by RT-PCR was used to genotype EV as a routine detection method, and real-time RT-PCR detection of EV71 was used as the standard detection method since 2008, providing more rapid analysis [22]. The partial *VP1* sequences of EV71 have been deposited in GenBank with the accession numbers EU729114, EU729123, AY125972, HM443644, HM44366, HM443645-HM443662, KC162863-KC162883 and KT182938-KT182945.

### Phylogenetic analysis

To analyze the genetic characteristics of EV71 cases, the *VP1* gene from EV71 isolates was examined by conventional RT-PCR. *VP1* partial sequences for the EV71 isolates were compared with those of foreign strains. Multiple sequence alignments with the respective reference strain sequences were carried out using DNAstar Sequence Alignment Editor. MEGA software (version 4.0) was used for phylogenetic analysis. Phylogenetic trees were constructed using the neighbor-joining method (bootstrap resampling of 1000 replicates). Sequence data from both strands were aligned and edited using MEGA.

## Results

### The prevalence and epidemiological features of EV71 infections

From 2007 to 2012, we collected specimens and clinical information from 9987 patients with suspected EV infections in the ROK. The annual outcomes of EV and EV71 from 2007 to 2012 are shown in Table 1. A total of 3502 (35 %) patients were confirmed as EV positive by laboratory analyses. EV71 infection was confirmed 585 (16.7 %) of EV-positive patients (Table1). In the ROK, EV71 was detected for the first time in 2000 (12 cases), and no cases were confirmed from 2001 to 2005 (data not shown). In 2006, six cases were confirmed as EV71. Since 2009, EV71 has been confirmed at a frequency similar to those of other EV types (Table 1).

**Table 1:** Prevalence of enterovirus71 and enterovirus in the Republic of Korea from 2007 to 2012

Year	No. samples	No. (%) EV positive	No. (%) EV71	Rank^a^
2007	691	132 (19.1)	22 (16.7)	3^b^
2008	1911	1010 (52.9)	2 (0.2)	Out of rank 5^c^
2009	2765	869 (31.4)	127 (14.6)	1
2010	1445	557 (38.5)	190 (34.1)	1
2011	1812	593 (32.7)	118 (19.9)	1
2012	1363	341 (25.0)	126 (37.0)	1
Total	9987	3502 (35.1)	585 (16.7)	-

*EV* enterovirus

^a^Most frequent ranking among human enterovirus genotype

^b^Coxsackie virus B2, Coxsakievirus A9 was rank1 and 2 in 2007

^c.^Included Echovirus30, Echovirus6, CoxsakievirusA10, CoxsakievirusB3, CoxsakievirusB1 in rank 5

The overall age distribution of enrolled patients infected with EV71 included neonates through patients who were 37 years of age (data not shown). The mean age was 3.57 ± 4.6 years, and median age was 3 years. During this period, most patients were less than 15 years of age (95.4 %); in particular, 476 (81.4 %) cases occurred in young children less than 5 years age. Nine (1.6 %) neonates (less than 1 month old) and 178 (30.3 %) children less than 1 year of age acquired EV71 infections. Among the study population, age was not listed for 16 (3.2 %) cases. Of the EV71 isolates, 339 were from males and 235 were from females, giving a male-to-female ratio of approximately 3:2 (data not shown). Information regarding the sex of 11 patients was unavailable.

**Fig. 1:**
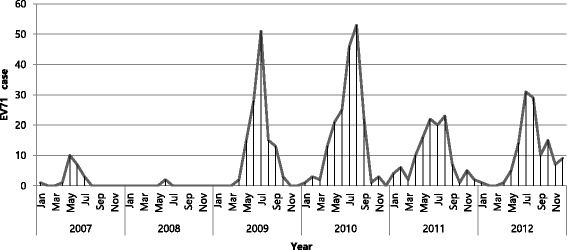
Seasonal distribution of EV71 infection from 2007 to 2012 in the Republic of Korea. EV71, enterovirus 71

In this study, monthly distribution showed that EV71 infection followed an obvious seasonal pattern and occurred during the short period from June to August in 2007–2012 (Fig. 1).

### Clinical features of patients with EV71

The classification for clinical manifestations of EV71-positive patients is described in Table 2. HFMD was the major clinical sign (*n* = 364, 62.2 %). We found that 198 cases exhibited HFMD only, while 166 (28.4 %) cases exhibited HFMD with CNS complications. The incidence of EV71infection of the CNS accompanied by HFMD was increased beginning in 2009 (no cases in 2008 and 26.0 % of patients in 2009). The other clinical symptoms were aseptic meningitis (*n* = 136, 23.2 %), encephalitis or meningoencephalitis without HFMD (*n* = 19, 3.2 %), myocarditis (*n* = 2, 0.3 %), and sepsis (*n* = 13, 2.2 %). We also received reports of death (*n* = 5, 1.1 % among 435 cases during 2009–2011) associated with EV71 infection between 2009 and 2011 (two cases in 2009, two cases in 2010 and one case in 2011). Fatalities occurred due to neurological complications. In 2009, two deaths caused by EV71 infection occurred in female infants approximately 12 months of age who showed signs of encephalitis accompanied by HFMD. Additionally, in 2010, two male infants died of EV71 infection at 10 and 13 months of age. One patient showed signs of meningitis accompanied by HFMD, while other patients suffered from lung hemorrhage with HFMD in 2010. Another case of EV71 infection occurred in a 16-month-old infant with myocarditis and meningitis accompanied by HFMD in 2011.

**Table 2:** Clinical manifestationsfromcases of enterovirus71 infection in Korea between 2007 and 2012

Clinical syndrome/Year	2007		2008		2009		2010		2011		2012		Total	
	*n*	%	*n*	%	*n*	%	*N*	%	*N*	%	*n*	%	*n*	%
HFMD or herpangina	11	50.0	1	50.0	103	81.1	133	70.0	58	49.2	58	46.0	364	62.2
Without complication	11	50.0	1	50.0	70	55.1	70	36.8	10	8.5	36	28.6	198	33.8
With aseptic meningitis					33	26.0	63	33.2	42	35.6	18	14.3	156	26.7
With encephalitis or ME									6	5.1	3	2.4	9	1.5
With ADEM											1	0.8	1	0.2
Aseptic meningitis only	8	36.4			18	14.2	46	24.2	31	26.3	33	26.2	136	23.2
Encephalitis or ME only					2	1.6	6	3.2	7	5.9	4	3.2	19	3.2
Myocarditis									2	1.7			2	0.3
Sepsis					1	0.8	3	1.6	5	4.2	4	3.2	13	2.2
Others^a^	3	13.6	1	50.0	3	2.4	2	1.1	15	12.7	27	21.4	51	8.7
Total	22	3.8	2	0.3	127	21.7	190	32.5	118	20.2	126	21.5	585	100.0
Death					2	1.6	2	1.1	1	0.8			5	0.9

*HFMD* hand-foot-mouth disease, *ADEM* acute disseminated encephalomyelitis, *ME* meningoencephalitis

^a^Symptoms such as fever, cold syndrome, abdominal pain, sore throat and manifestations of a suspected enterovirus infection

### Sequence comparison and phylogenetic analysis of EV71

**Fig. 2:**
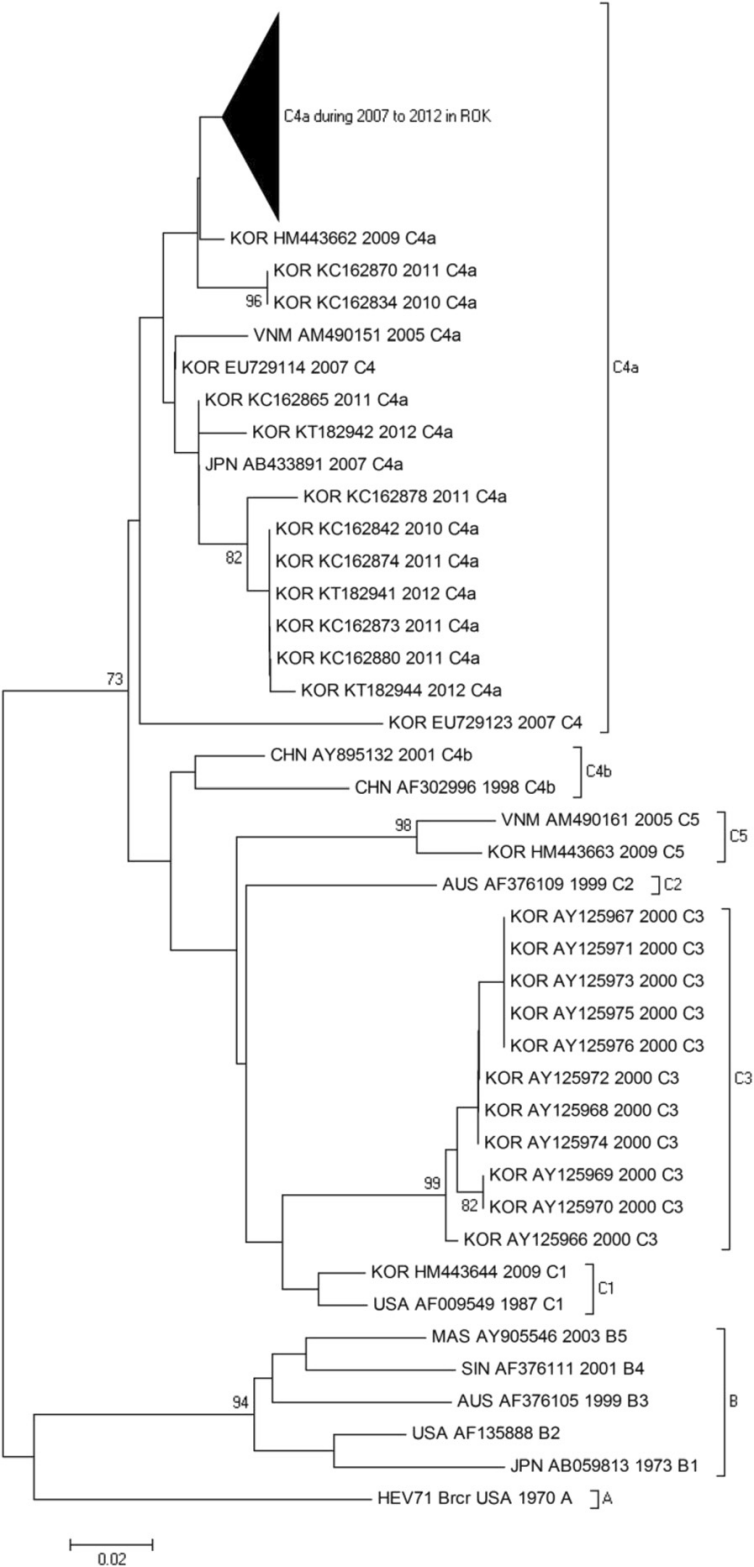
Phylogenetic dendrogram based on the partial nucleotide sequence of the *VP1* gene using the neighbor-joining method (bootstrapped 1000 times). The scale bar represents 2 % difference in nucleotide sequences. GenBank accession numbers are given behind the country name

EV71 genetic groups can be classified into three distinct clusters (A, B and C) with some temporal and regional subclustering [7]. Based on this classification, all of the isolates in the current study, with the exception of two strains (HM443644-2009 typed as C1 and HM443663-2009 typed as C5), belonged to subgenotype C4a (Fig. 2). These strains (i.e., C4a) are common in China, Japan and Vietnam [7].

## Discussion

In this study, we examined the occurrence of EV71 infection during the period from 2007 to 2012 according to the National EV Surveillance System in the ROK. The present study included clinical records of EV71-infected patients and analysis of laboratory findings, including determination of genetic differences between outbreaks in the ROK and those in other countries. Moreover, in this study, we found that EV71 infection exhibited a seasonal trend based on epidemiological information for EV71 infection and focused on EV71 infection as an etiological risk factor associated with complications.

We found that this virus tended to infect younger individuals and caused more severe symptoms, including death, in very young patients. EV71 infection, which was the most frequently detected strain during the study period, is associated with neurological complications and, since 2009, has been responsible for most cases of EV infection. Notably, EV71 was not detected at all between 2001 and 2005, after 12 cases were reported in 2000 [19].

EV71 induces HFMD or herpangina, which can exhibit a severe clinical course accompanied by neurological complications, leading to death in five cases. EV71 was reported as a cause of HFMD, which is associated with a high prevalence of acute neurologic disease [23]. Since 1997, multiple cases of EV71 infection have been shown to be associated with severe aseptic meningitis and pulmonary edema in the Asia-Pacific region, including Taiwan, Malaysia, Singapore and Japan [12, 24–27]. Moreover, HFMD outbreaks in this region included the ROK have been shown to be associated with EV71 infection and continue to pose an enormous threat to public health due to the increased morbidity and mortality rates associated with HFMD-related neurological complications.

Based on phylogenetic analyses, the ROK isolates belonged to a cluster of the subtype C4a and showed high homology with isolates circulating in other countries. EV71 often occurs in a cyclical pattern, emerging every 2–3 years, in various countries [10]. Interestingly, a C4 virus has been the predominant circulating strain in China since 1998, Taiwan since 2002 and Japan since 2003 [7, 8]. Thus, EV71 subtype C4 has become a predominant strain and has been continuously circulating and causing epidemics in neighboring countries and regions. Moreover, the C4a subtype of EV71 was dominantly identified in the ROK since 2009 and has been continuously monitored for more than 4 years. Previous studies have suggested that the EV71 cyclical pattern may arise according to the proportion of herd immunity among the susceptible population after a major outbreak [28, 29].

The clinical manifestations of EV71 infection were consistent with results from previous studies and showed that the majority of cases occurred in children less than 5 years of age, as has been observed in other countries [30, 31]. Thus, infection with EV71 may become serious public health concern, particularly for children. Additionally, EV71 infections generally exhibit an increased incidence in summer and early autumn [30, 32]. As expected in temperate climates, the present study revealed a seasonal pattern of distribution, with transmission peaking in the summer and decreasing in the period from autumn to spring. Few cases occurred during the winter.

One limitation of this study was that only a subset of *VP1* sequences were used for phylogenetic studies. Another study found that the results of *VP1*-based phylogenetic trees are not similar to those of complete genome-based analyses [33]. Published data on EV epidemiology revealed that recombination between EV71 genotypes B or C and other types of coxsakieviruses occurred in Asian-Pacific countries [8, 9, 11, 15, 34–36]. For EV, genetic recombination has been reported to result in the emergence of viruses with altered pathogenic potential [36]. Unfortunately, in this study, we were unable to examine the occurrence of homologous recombination. Further studies are needed to analyze EV71 dynamic mutations and genetic variation in the EV71 subtype through the analysis of full genome sequences.

## Conclusions

The present study indicated that EV71 was prevalent among EV-positive patients in the ROK during a recent 6-year period. Because of the impact of EV71 infection on public health, HFMD was designated as a nationally notifiable communicable disease in 2009, allowing for more comprehensive surveillance programs in the ROK. The surveillance reported in this study provides valuable data on the phylogenetic patterns and clinical manifestations associated with EV71 infection. Our findings also provide relevant information for future development of preventive vaccines and treatment plans. In addition, the information on EV71 infection reported in this study may provide important insights into the general outcomes of EV71 infection and associated acute neurologic disease.

## Abbreviations

CNS, central nervous system; CSF, cerebrospinal fluid; EV, enterovirus; HFMD, hand, foot and mouth disease; KCDC, Korea Centers for Disease Control and Prevention; ROK, Republic of Korea; RT-PCR, reverse transcription polymerase chain reaction; VP, virus capsid protein
